# Core-Shell Fe_3_O_4_@C Nanoparticles as Highly Effective T_2_ Magnetic Resonance Imaging Contrast Agents: In Vitro and In Vivo Studies

**DOI:** 10.3390/nano14020177

**Published:** 2024-01-12

**Authors:** Huan Yue, Dejun Zhao, Tirusew Tegafaw, Mohammad Yaseen Ahmad, Abdullah Khamis Ali Al Saidi, Ying Liu, Hyunsil Cha, Byeong Woo Yang, Kwon Seok Chae, Sung-Wook Nam, Yongmin Chang, Gang Ho Lee

**Affiliations:** 1 Department of Chemistry, College of Natural Sciences, Kyungpook National University, Taegu 41566, Republic of Korea; yuehuan888@gmail.com (H.Y.); djzhao.chem@gmail.com (D.Z.); tirukorea@gmail.com (T.T.); yaseen.knu@gmail.com (M.Y.A.); aalsaidi@knu.ac.kr (A.K.A.A.S.); ly1124161@gmail.com (Y.L.); 2 Division of Biomedical Science, School of Medicine, Kyungpook National University, Taegu 41944, Republic of Korea; hyunsil901002@gmail.com; 3 Theranocure, Medlifescience Bldg. 1, Chilgok, Bukgu, Taegu 41405, Republic of Korea; byungwoo1128@naver.com; 4 Department of Biology Education, Teachers’ College, Kyungpook National University, Taegu 41566, Republic of Korea; kschae@knu.ac.kr; 5 Department of Molecular Medicine, School of Medicine, Kyungpook National University, Taegu 41944, Republic of Korea; nams@knu.ac.kr

**Keywords:** Fe_3_O_4_@C nanoparticle, core shell, magnetic resonance imaging, contrast agent, highly effective

## Abstract

Magnetite nanoparticles (Fe_3_O_4_ NPs) have been intensively investigated because of their potential biomedical applications due to their high saturation magnetization. In this study, core–shell Fe_3_O_4_@C NPs (core = Fe_3_O_4_ NPs and shell = amorphous carbons, d_avg_ = 35.1 nm) were synthesized in an aqueous solution. Carbon coating terminated with hydrophilic –OH and –COOH groups imparted excellent biocompatibility and hydrophilicity to the NPs, making them suitable for biomedical applications. The Fe_3_O_4_@C NPs exhibited ideal relaxometric properties for T_2_ magnetic resonance imaging (MRI) contrast agents (i.e., high transverse and negligible longitudinal water proton spin relaxivities), making them exclusively induce only T_2_ relaxation. Their T_2_ MRI performance as contrast agents was confirmed in vivo by measuring T_2_ MR images in mice before and after intravenous injection.

## 1. Introduction

Magnetite nanoparticles (Fe_3_O_4_ NPs) have attracted considerable attention in many application areas, including environmental treatment [1,2,3], food analysis [4,5,6], biosensors [7,8], protein immobilization [9], targeted drug delivery [10,11,12,13,14], separation [15,16,17], magnetic resonance imaging (MRI) [18,19,20,21,22,23,24,25,26,27], magnetofection [28,29], tissue engineering [30], and hyperthermia [31,32]. In particular, their good biocompatibility, as verified by the U.S. Food and Drug Administration [33], facilitates their commercialization.

The high attraction of Fe_3_O_4_ NPs originates from their excellent magnetic properties and good biocompatibility [34]. However, their high surface energy and dipole–dipole interaction make them unstable and prone to aggregation [35]. Consequently, the modification of their surfaces is essential for biomedical applications. They can achieve this goal using protective and non-magnetic shell coating [36,37]. Carbon coating terminated with hydrophilic functional groups is a promising surface-coating option because carbon is nontoxic and chemically inert. 

The saturation magnetization (M_s_) of Fe_3_O_4_ NPs increases with an increasing particle size toward the bulk value (93–98 emu/g) [38,39,40]. Consequently, their relaxometric properties become more favorable to transverse (T_2_) water proton spin relaxation induction than to longitudinal (T_1_) water proton spin relaxation induction with increasing particle size. Under such conditions, they can exclusively induce only the T_2_ water proton spin relaxation, making them function as highly effective T_2_ MRI contrast agents. 

Herein, we synthesized large Fe_3_O_4_ NPs for use as T_2_ MRI contrast agents. We followed our former strategy of coating carbon on the Fe_3_O_4_ NPs via the carbonization of dextrose in aqueous media in the presence of Fe_3_O_4_ NPs [36,37]. This process leaves a large amount of –OH and some –COOH groups on the terminal carbon shells, allowing for good colloidal stability of the resulting carbon-coated Fe_3_O_4_ NPs (i.e., core–shell Fe_3_O_4_@C NPs) in aqueous media. However, there exist only a few studies on water proton spin relaxivities [41] and in vivo MRI [42] of Fe_3_O_4_@C NPs. Herein, the magnetic properties, water proton spin relaxivities, in vitro cellular cytotoxicity, and in vivo T_2_ MR images were obtained to demonstrate the potential of the Fe_3_O_4_@C NPs as contrast agents in T_2_ MRI. We found that the synthesized Fe_3_O_4_@C NPs are highly effective T_2_ MRI contrast agents based on their ideal relaxometric properties for T_2_ MRI contrast agents (i.e., high transverse (r_2_) and negligible longitudinal (r_1_) water proton spin relaxivities). We found that the Fe_3_O_4_@C NPs were very effective in liver imaging as T_2_ MRI contrast agents. 

## 2. Materials and Methods

### 2.1. Chemicals

FeSO_4_·7H_2_O (≥99%), NaOH (>99.99%), and dextrose (C_6_H_12_O_6_) (>99.5%) were purchased from Sigma-Aldrich (St. Louis, MO, USA), and used as received. Triple-distilled water was used for washing the NPs and preparing the NP colloidal solution. 

### 2.2. Synthesis

#### 2.2.1. Synthesis of the Fe_3_O_4_ NPs

**Figure 1:**
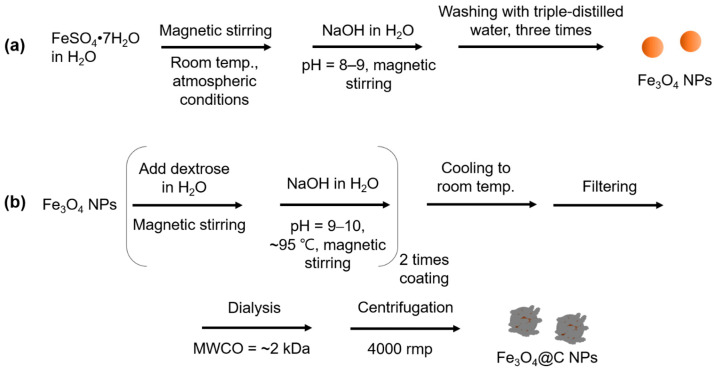
Facile synthesis of (**a**) the Fe_3_O_4_ NPs and (**b**) the Fe_3_O_4_@C NPs in an aqueous solution.

First, 0.5 mmol of FeSO_4_·7H_2_O was added into a 50 mL beaker containing 20 mL of triple-distilled water at room temperature (22 °C) with magnetic stirring under atmospheric conditions (Figure 1a). Then, 10 mmol of NaOH dissolved in 20 mL of triple-distilled water was gradually added to the aforementioned solution to obtain a pH of 8–9 with continuous magnetic stirring for 30 min. The solution was transferred to a 500 mL beaker containing 400 mL of triple-distilled water and then magnetically stirred for 0.5 h. The product solution was placed in a refrigerator (~5 °C) to obtain the Fe_3_O_4_ NP precipitation. The top solution containing Na^+^, OH^−^, and unreacted precursors was removed, and the remaining Fe_3_O_4_ NPs were washed thrice using the same process. 

#### 2.2.2. Synthesis of the Core-Shell Fe_3_O_4_@C NPs

The previously obtained Fe_3_O_4_ NPs were added into a 100 mL three-necked round-bottom flask containing 1 mmol of dextrose in 10 mL of triple-distilled water at room temperature with magnetic stirring under atmospheric conditions (Figure 1b). After 30 min of magnetic stirring, 4 mmol of NaOH dissolved in 5 mL of triple-distilled water was gradually added to the aforementioned solution to make the solution pH 9–10, followed by 2 h of magnetic stirring at ~95 °C to coat the Fe_3_O_4_ NPs with amorphous carbon via carbonization of dextrose. This coating process was repeated twice to increase the coating thickness. The reaction solution temperature decreased to room temperature and the solution underwent filtering using Whatman filter paper. Na^+^, OH^−^, and free dextrose were removed via dialysis (MWCO = ~2 kDa) using 1 L of deionized water with magnetic stirring for 3 days, and the outside water solution was replaced thrice with deionized water. After dialysis, free carbon NPs were removed through decantation of the supernatant solution following centrifugation at 4000 rpm for 0.7 h (VS-4000 N, Vision Scientific Co., Ltd, Bucheon, Republic of Korea). This process was repeated thrice following the redispersion of the settled NPs in the centrifuge tube with triple-distilled water. The final Fe_3_O_4_@C NP solution was split into two portions. One portion was used for the NP solution sample preparation in triple-distilled water, and the other portion was lyophilized in vacuum to obtain powder sample. 

### 2.3. Characterization

The average particle diameter (d_avg_) and morphology of the Fe_3_O_4_@C NPs were measured using high-resolution transmission electron microscopy (HRTEM) (Titan G2 ChemiSTEM CS Probe, FEI, Hillsboro, OR, USA). Two drops of the aqueous solution sample were placed on a 200-mesh copper grid covered with a carbon film (PELCO no.160, Ted Pella, Inc., Redding, CA, USA), followed by drying in air. High-angle annular dark-field scanning transmission electron microscopy (HAADF-STEM) images with elemental mapping were obtained to confirm the carbon coating. The Fe-concentration in the aqueous solution sample was estimated using inductively coupled plasma-atomic emission spectroscopy (Optima 7300DV and Avio500, Perkin Elmer, Waltham, MA, USA). Zeta potential (ζ) was measured (Malvern Panalytical, Zetasizer Nano ZS, Westborough, MA, USA) using a diluted solution sample. The crystal structure of the powder samples was measured using a multi-purpose X-ray diffraction (XRD) spectrometer (X’PERT PRO MRD, Philips, Almelo, The Netherlands) equipped with unfiltered CuKα radiation (λ = 0.154184 Å) using a scan range of 2θ = 15–100° and a scan step of 2θ = 0.03°. The carbon coating on the Fe_3_O_4_ NP surfaces was investigated by recording the Fourier transform infrared (FT-IR) absorption spectra (Galaxy 7020A, Mattson Instruments, Inc., Madison, WI, USA) using the power sample in KBr as pellets. The relative amounts of carbon coating and Fe_3_O_4_ NPs were assessed through thermogravimetric analysis (TGA) (SDT-Q6000, TA Instruments, New Castle, DE, USA). In addition, the carbon-coating composition (C/H/O) and amount were estimated via elemental analysis (EA; Flash 2000, ThermoFisher, Waltham, MA, USA). The magnetic properties of the powder sample (20–30 mg) were characterized by recording a magnetization (M)-applied field (H) curve (−2.0 T ≤ H ≤ 2.0 T) at 300 K using a vibrating sample magnetometer (7407-S, Lake Shore Cryotronics Inc., Westerville, OH, USA). The mass-corrected magnetization (i.e., net M) of the core Fe_3_O_4_ NPs without surface-coating carbon shell was obtained based on the net mass of the Fe_3_O_4_ NPs obtained from the TGA curve. 

### 2.4. Water Proton Spin Relaxivities and Map Images

The T_1_ and T_2_ water proton spin relaxation times and longitudinal (R_1_) and transverse (R_2_) map images were measured at 22 °C using a 3-T MRI scanner (MAGNETOM Trio Tim, Siemens, Munchen, Bayern, Germany). The original sample solution was diluted with triple-distilled water to obtain diluted NP colloidal solutions with 0.1, 0.05, 0.025, 0.0125, and 0.00625 mM [Fe]. T_1_ relaxation times were measured using an inversion recovery method, while T_2_ relaxation times were obtained using multiple spin-echo appraisal with the Carr–Purcell–Meiboom–Gill pulse sequence. The r_1_ and r_2_ values were obtained from the linear fitting plot slopes of 1/T_1_ and 1/T_2_ inverse relaxation times versus Fe concentration, respectively. 

### 2.5. In Vitro Cellular Cytotoxicity

A Luminescent Cell Viability Assay kit (CellTiter-Glo, Promega, Madison, WI, USA) was used for in vitro cellular cytotoxicity estimation of the NPs. Adenosine triphosphate was detected using a luminometer (Victor 3, Perkin Elmer, Waltham, MA, USA). Human prostate cancer (DU145) and normal mouse hepatocyte (NCTC1469) cell lines were used for the tests. The cells were seeded onto a 24-well cell culture plate and incubated for 24 h (5 × 10^4^ cell density, 500-μL cells per well, 5% CO_2_, and 37 °C). Five test NP colloidal solutions (with 10, 50, 100, 200, and 500 μM [Fe]) were prepared via dilution of the NP colloidal solution with a sterile phosphate buffer saline solution, from which 2 μL was taken out to drop onto the cells, followed by incubation for 48 h. The cell viability was determined, followed by normalization with respect to those of the control cells with 0 mM [Fe]. The average cell viability was obtained via three measurements. 

### 2.6. In Vivo MRI Experiments

All in vivo mouse experiments were authorized by the Animal Research Committee of Kyungpook National University and conducted in accordance with its rules and guidelines. A 3-T MRI scanner was used to obtain T_2_ MR images. The 1.5% isoflurane in oxygen was used for anesthetization of ICR (Institute of Cancer Research, London, UK) mice (~30 g) for imaging. Two mice (N = 2) were used. MR image acquisition was conducted prior to and following intravenous injection (IV) of the sample solution into the mice tails at an injection dose of 0.05 mmol Fe per kg mice. A warm water blanket was used to maintain the mice’s body temperature at ~37 °C during imaging. The T_2_ MR images were obtained using the following parameters: MR field = 3 T, number of slices = 11, number of acquisitions = 2, field of view (FOV) = 10 cm, phase FOV = 1, spacing gap = 0.1 mm, frequency = 300 Hz, phase = 300, temperature = 37 °C, slice thickness = 1.5 mm, repetition time (TR) = 4300 ms, and echo time (TE) = 45 ms. 

## 3. Results

### 3.1. Particle Diameter and Crystallinity of the Fe_3_O_4_@C NPs

**Figure 2:**
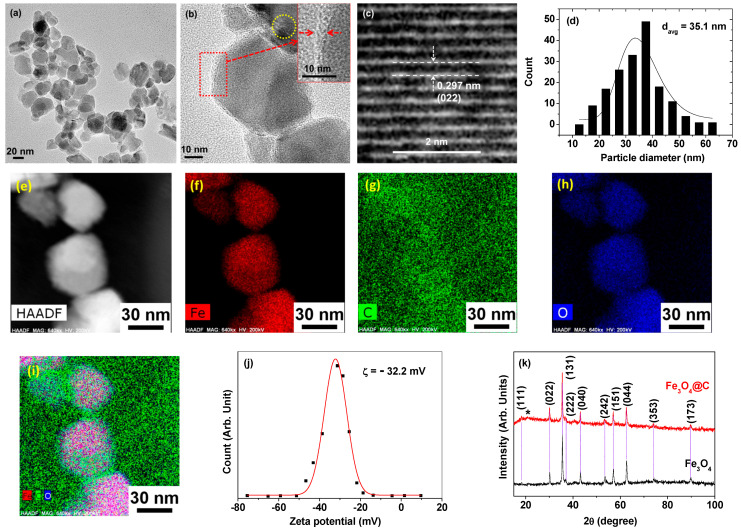
(**a**) HRTEM image. (**b**) Magnified HRTEM image: the dotted square is further magnified on the top right to show carbon-coating shell thickness, labeled with two arrows. Dotted circle is further magnified in (**c**) to show (022) plane lattice fringes. (**d**) Particle diameter distribution of the Fe_3_O_4_@C NPs obtained from the HRTEM images and the log-normal function fitting curve. (**e**) HAADF-STEM image of the Fe_3_O_4_@C NPs and elemental mapping of (**f**) Fe (red), (**g**) C (green), (**h**) O (blue), and (**i**) merged elements. (**j**) Zeta potential curve in the aqueous solution. (**k**) XRD patterns of the Fe_3_O_4_ and Fe_3_O_4_@C NPs: all XRD peaks (indicated with vertical dotted lines) were assigned based on (hkl) Miller indices matching FCC Fe_3_O_4_. The broad peak at 2θ = ~21° (labeled as *) for the Fe_3_O_4_@C NPs is due to amorphous carbon.

The particle diameter of the Fe_3_O_4_@C NPs was estimated from the HRTEM images (Figure 2a,b), and a core-shell structure with a carbon shell at the border of the Fe_3_O_4_ NPs was observed in the magnified HRTEM image (Figure 2b). Clear lattice fringes of the core Fe_3_O_4_ NP with an interplanar distance of d = 0.297 nm, corresponding to the (022) planes of cubic Fe_3_O_4_, were observed (Figure 2c), confirming the successful synthesis of the Fe_3_O_4_ NPs. The d_avg_ of the Fe_3_O_4_@C NPs of 35.1 ± 1.4 nm (Table 1) was estimated from the log-normal function that fit to the observed particle diameter distribution (Figure 2d). To further confirm the existence of the surface carbon shell, elemental mapping of C, Fe, and O in the Fe_3_O_4_@C NPs was performed. Figure 2e–i show the HAADF-STEM images of the Fe_3_O_4_@C NPs and the elemental mapping of Fe (red), O (blue), and C (green). A higher intensity of carbon on the core Fe_3_O_4_ NPs compared with that of background carbon film was observed (Figure 2i), confirming the successful carbon-shell coating on the Fe_3_O_4_ NP surfaces. The observed high zeta potential (ζ = −32.2 mV) of the Fe_3_O_4_@C NPs in aqueous solution confirmed their good colloidal stability (Figure 2j and Table 1). 

The XRD patterns of the Fe_3_O_4_ and Fe_3_O_4_@C NPs (Figure 2k) exhibited clear sharp peaks matching the cubic Fe_3_O_4_ reference pattern [43], demonstrating the successful synthesis of the face-centered cubic (FCC) Fe_3_O_4_ NPs. The cell constant was estimated to be 8.396 Å, consistent with the reported value of 8.3963 ± 0.0005 Å [43]. A broad peak at 2θ = ~21° (labeled as *) for the Fe_3_O_4_@C NPs, which did not appear for the Fe_3_O_4_ NPs, is due to amorphous carbon [44], confirming the successful carbon coating on Fe_3_O_4_ NP surfaces. 

### 3.2. Surface-Coating Analysis

**Figure 3:**
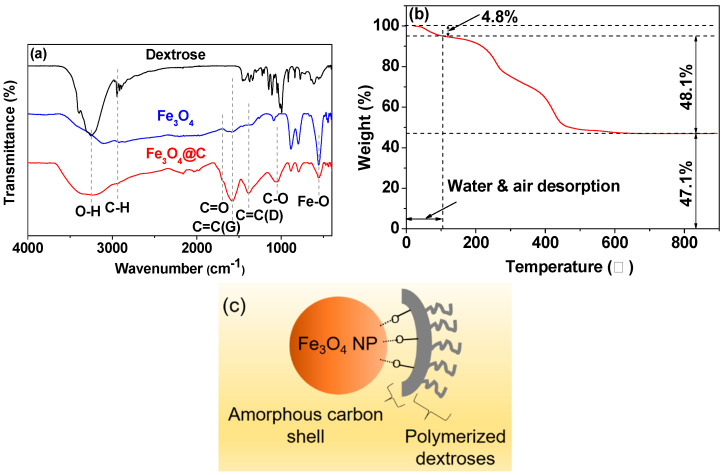
(**a**) FT-IR absorption spectra: Fe_3_O_4_@C NPs (bottom), Fe_3_O_4_ NPs (middle), and dextrose (top). (**b**) TGA curve of the Fe_3_O_4_@C NPs under air flow. (**c**) Proposed core-shell structure of the Fe_3_O_4_@C NPs.

To investigate the carbon coating, the FT-IR absorption spectra (Figure 3a) of the Fe_3_O_4_ NPs, the Fe_3_O_4_@C NPs, and dextrose pelletized in KBr were recorded. Two specific peaks at 1384 and 1578 cm^−1^ in the FT-IR absorption spectrum of the Fe_3_O_4_@C NPs, corresponding to C=C (D) and C=C (G) vibrations [45] (Table 2), respectively, were observed, supporting the successful amorphous carbon coating on the Fe_3_O_4_ NP surfaces. These two peaks do not exist in the spectrum of free dextrose because there is no C=C bonding in dextrose. The C=O stretching peak at 1699 cm^−1^ confirmed the existence of –COOH groups on the carbon-coating surfaces. The strong stretching peaks of O–H at 3248 cm^−1^ and C–O at 1048 cm^−1^ confirmed the presence of a large amount of –OH in the carbon-coating shell. The existence of these two hydrophilic groups supports the observed high zeta potential and good colloidal stability in aqueous media. Note that the observed O–H peak is different from the H–O–H stretching peak of water at 3398 cm^−1^. The Fe_3_O_4_ NPs in the sample were supported by the strong Fe–O stretching vibration at 556 cm^−1^. 

The carbon coating amount in weight percent (wt.%) was estimated to be 48.1% from the TGA curve (Figure 3b and Table 1). The initial mass drop (4.8%) was due to water and air desorption from the sample. The remaining 47.1% after TGA was due to iron oxide NPs. In addition, the carbon coating wt.% including water and air was estimated to be 58.04% (Table 1) using EA by summing the wt.% of C/H/O = 28.90/3.11/26.03, and the remaining mass of 41.96% was due to the Fe_3_O_4_ NPs. Owing to the high melting point of Fe_3_O_4_, i.e., 1577 °C, the Fe_3_O_4_ NPs will not decompose during EA. Therefore, the oxygen from EA exists mainly due to the surface-coating carbon layer with the minor contribution from air and water, which are contained in the surface-coating carbon layer. The C/H/O molar ratio of 1.48/1.91/1.00 estimated from the aforementioned results supports the existence of a large amount of aliphatic carbon and hydroxyl groups, as observed in the FT-IR absorption spectrum, because fully carbonized carbon does not contain H and O. When considering the C/H/O (1/2/1) molar ratio of dextrose, the 1.48 molar ratio of C/O in the sample was only raised by 0.4 from 1.0 of dextrose, supporting the incomplete carbonization of dextrose (i.e., not infinite C/O ratio). Therefore, the fully carbonized amorphous carbon amount was roughly estimated to be 32.43% (= 0.48/1.48), and the remaining 67.57% was the incompletely carbonized dextrose (or polymerized dextrose, because carbonization proceeds through dextrose polymerization [46,47]). The core-shell structure of the Fe_3_O_4_@C NPs, showing the outer polymerized dextroses with a large number of –OH groups and the inner amorphous carbon shell covering the core Fe_3_O_4_ NP surfaces, is presented based on this in Figure 3c. 

### 3.3. Magnetic Property

**Figure 4:**
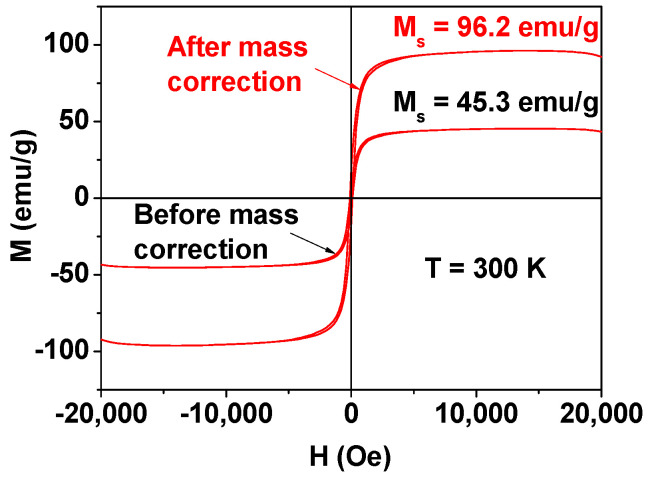
M–H curves of the Fe_3_O_4_@C NPs at 300 K before and after mass correction.

Figure 4 exhibits the M–H curves of the Fe_3_O_4_@C NPs before and after mass correction. By using the estimated net Fe_3_O_4_ amount from the TGA data, the mass-corrected (i.e., net) magnetization (M) of the Fe_3_O_4_ NPs was 96.2 emu/g, which was similar to the bulk value of 93–98 emu/g [38,39,40]. 

### 3.4. In Vitro Cellular Cytotoxicity

**Figure 5:**
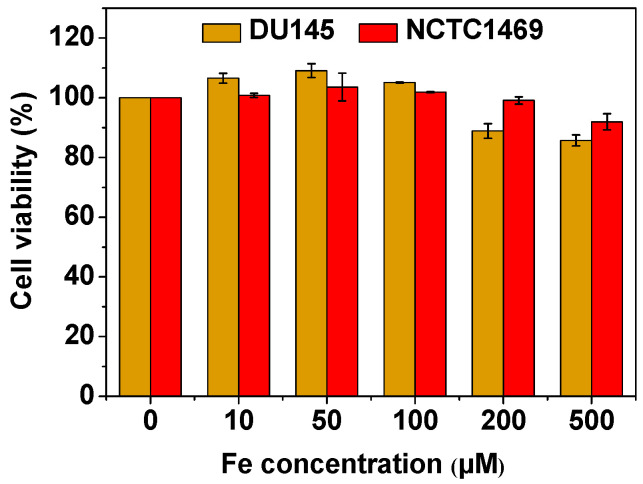
In vitro cell viability of the DU145 and NCTC1469 cells after incubation with the Fe_3_O_4_@C NPs up to 500 μM [Fe].

Because of the good biocompatibility of Fe_3_O_4_ and amorphous carbon, good cell viability (>85%) was observed in the cell viability assay of the DU145 and NCTC1469 cells up to 500 μM [Fe] (Figure 5). In addition, the good biocompatibility of the Fe_3_O_4_@C NPs was confirmed from mice survival following the in vivo MRI experiments. 

### 3.5. r_1_ and r_2_ Values and R_1_ and R_2_ Map Images

**Figure 6:**
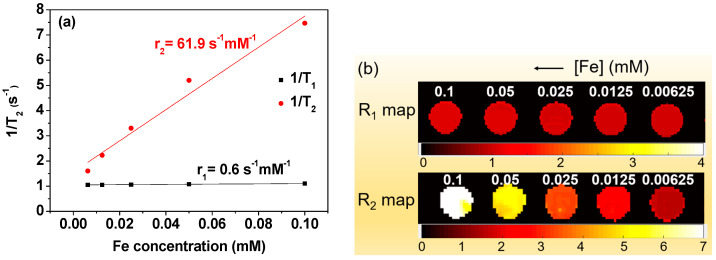
(**a**) Plots of 1/T_1_ and 1/T_2_ inverse relaxation times versus Fe concentration. The slopes correspond to the r_1_ and r_2_ values, respectively. (**b**) R_1_ and R_2_ map images as a function of Fe concentration, showing clear dose-dependent contrast enhancement in the R_2_ map images but negligible dose-dependent contrast enhancement in the R_1_ map images; the scale unit is s^−1^.

To characterize the potential of the Fe_3_O_4_@C NPs as T_2_ MRI contrast agents, their r_1_ and r_2_ values were estimated from the linear fitting plots of 1/T_1_ and 1/T_2_ inverse relaxation times versus Fe concentration (Figure 6a). The high r_2_ (=61.9 s^−1^ mM^−1^) and very low r_1_ (=0.6 s^−1^ mM^−1^) values (Table 1) indicate that the Fe_3_O_4_@C NPs can exclusively induce only T_2_ water proton spin relaxation with minimal induction of T_1_ water proton spin relaxation. This was clearly visualized in the R_1_ and R_2_ map images, in which the evidently clear dose-dependent contrast enhancement was observed only in the R_2_ map images whereas no such contrast enhancement was observed in the R_1_ map images (Figure 6b). Note that as the Fe concentration increased, the color in map images was made to become whiter in both R_1_ and R_2_ map images. 

### 3.6. In Vivo T_2_ MR Images

**Figure 7:**
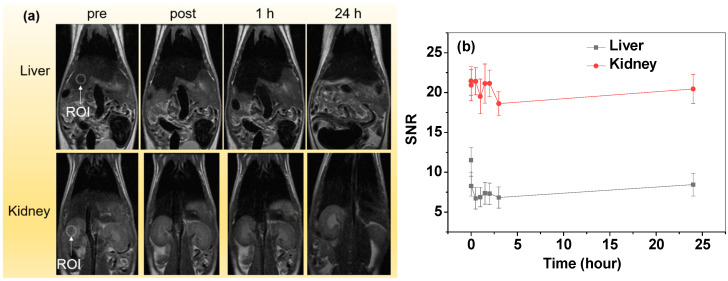
(**a**) In vivo coronal T_2_ MR images of the liver and kidneys of the mice (dotted circles indicate ROIs used for estimating SNRs) as a function of time (“pre” and “post” indicate “just before” and “just after” IV injection, respectively). (**b**) SNR-ROI plots as functions of time.

The in vivo T_2_ MR images were obtained at different capture times up to 24 h (Figure 7a). To show, quantitatively, the contrast change with time, the signal-to-noise ratio (SNR) of the region-of-interest (ROI) in the liver was measured and plotted as a function of time (Figure 7b). The plot clearly showed the negative enhancements of the liver with time. On the contrary, the negative contrast enhancements of the kidneys were small (Figure 7a,b). Therefore, the Fe_3_O_4_@C NPs are more appropriate for liver imaging. 

## 4. Discussion

The amorphous carbon coating in aqueous media via the carbonization of hydrophilic organic molecules in the presence of NPs is a promising surface coating method for biomedical applications of NPs, as demonstrated in this study. The carbon-coating shell is hydrophilic because of hydrophilic –OH and –COOH groups on its shell terminal. It is nontoxic because carbon is nontoxic and chemically inert. In this study, dextrose was carbonized in the presence of Fe_3_O_4_ NPs in aqueous media to obtain core-shell Fe_3_O_4_@C NPs. The obtained Fe_3_O_4_@C NPs displayed good colloidal stability in solution and very low cytotoxicity in the DU145 and NCTC1469 cells. 

The Fe_3_O_4_@C NPs showed a high saturation magnetization (M_s_) (=96.2 emu/g) and low coercivity (H_c_) (~100 Oe), confirming that the Fe_3_O_4_@C NPs are ferrimagnetic rather than superparamagnetic at room temperature at the observed particle size (d_avg_ = 35.1 nm). This is because their d_avg_ is greater than the superparamagnetic diameter (D_sp_: ~25 nm) [48]. In addition, their M_s_ value was similar to the bulk value of 93–98 emu/g [38,39,40], implying that the core Fe_3_O_4_ NPs reached the bulk saturation magnetization. 

For ideal T_2_ MRI contrast agents, the r_2_ value should be very high and the r_1_ value should be negligible (<1.0 s^−1^ mM^−1^). This was observed in the Fe_3_O_4_@C NPs, with high r_2_ (=61.9 s^−1^ mM^−1^) and very low r_1_ (=0.6 s^−1^ mM^−1^) values (Table 1) resulting from their high M_s_ value. Under such conditions, only the T_2_ (i.e., negligible T_1_) water proton spin relaxation can be exclusively induced by the NPs, rendering high negative contrasts in MR images. They can exclusively provide negative contrast enhancements in MR images and thus function as highly effective T_2_ MRI contrast agents. Highly negative contrasts were observed in in vivo T_2_ MR images using the Fe_3_O_4_@C NPs, supporting this. 

The in vivo T_2_ MRI performance of the Fe_3_O_4_@C NPs was evaluated by measuring the T_2_ MR images based on the IV injection of the sample solution into mouse tails. After injection, the liver clearly exhibited negative contrast enhancements. The clear contrast enhancements just after injection (labeled as “post” in Figure 7a) indicate that the NPs easily circulate through the blood vessels, indicating that the nanoparticle size (d_avg_ = 35.1 nm) is suitable for in vivo imaging via IV injection. The clear negative contrast enhancements in the liver are a consequence of the accumulation of the Fe_3_O_4_@C NPs in the liver. This accumulation indicates that the Fe_3_O_4_@C NPs were mostly sequestered by phagocytic Kupffer cells in the normal reticuloendothelial system in the liver [49]. However, the negative contrast enhancements at the kidneys were small, indicating a small amount of NP accumulation at the kidneys. Therefore, the Fe_3_O_4_@C NPs are more appropriate for liver imaging. Noting the observed very high r_2_ and negligible r_1_ values, the Fe_3_O_4_@C NPs should be highly effective T_2_ MRI contrast agents for liver imaging. For the present experiment, we tested the Fe_3_O_4_@C NPs potential T_2_ MRI contrast agents. We will further apply the Fe_3_O_4_@C NPs to the detection of diseases such as in tumor detection using tumor-model mice in the future.

## 5. Conclusions

Amorphous carbon terminated with hydrophilic –OH and –COOH groups was coated on Fe_3_O_4_ NP surfaces, providing chemical stability, good colloidal stability, and excellent biocompatibility to the Fe_3_O_4_@C NPs. The obtained core-shell Fe_3_O_4_@C NPs (d_avg_ = 35.1 ± 1.4 nm) exhibited highly negative contrast enhancements of the mice liver in the T_2_ MR images after IV injection. This was due to their ideal relaxometric properties for their applications as T_2_ MRI contrast agents (r_2_ = 61.9 s^−1^ mM^−1^, r_1_ = 0.6 s^−1^ mM^−1^, and r_2_/r_1_ = 103.2), which indicate that they can exclusively induce only T_2_ water proton spin relaxation, making them function as highly effective T_2_ MRI contrast agents for liver imaging. 

## Figures and Tables

**Table 1:** Summarized properties of Fe_3_O_4_@C NPs.

d_avg_ (nm)	ζ (mV)	Carbon Coating (wt.%)		Saturation Magnetization (emu/g)	Relaxivity (s^−1^ mM^−1^)		
		TGA ^1^	EA ^2^		r_1_	r_2_	r_2_/r_1_
35.1 ± 1.4	−32.2	48.1	58.04	96.2	0.6	61.9	103.2

^1^ Water and air excluded. ^2^ Water and air included.

**Table 2:** Observed FT-IR absorption frequencies (cm^−1^).

Sample	O–H	C–H	C=O	C=O (D)	C=O (G)	C–O	Fe–O
Dextrose	3248	2943	-	-	-	1010	-
Fe_3_O_4_@C NP	3248	2943	1699	1384	1578	1048	556

## Data Availability

The data presented in this study are available on request from the corresponding authors.
